# Ecosystem design as an avenue for improving services provided by carbonate producing marine ecosystems

**DOI:** 10.7717/peerj.12785

**Published:** 2022-01-20

**Authors:** Hildegard Westphal, Gary N. Murphy, Steve S. Doo, Thomas Mann, Alexander Petrovic, Christiane Schmidt, Marleen Stuhr

**Affiliations:** 1 Leibniz Centre for Tropical Marine Research (ZMT), Bremen, Germany; 2 Geoscience Department, Universität Bremen, Bremen, Germany; 3 King Abdullah University of Science and Technology (KAUST), Thuwal, Saudi Arabia; 4 Bundesanstalt für Geowissenschaften und Rohstoffe (BGR), Hannover, Germany; 5 Inter-University Institute for Marine Sciences (IUI), Eilat, Israel; 6 Bar-Ilan University, Ramat Gan, Israel

**Keywords:** Ecosystem design, Coral reef islands, Coastal erosion, Sea-level change, Restoration, Coastal protection, Sequestration, Conservation, Carbonate production, Coral health

## Abstract

Ecosystem Design (ED) is an approach for constructing habitats that places human needs for ecosystem services at the center of intervention, with the overarching goal of establishing self-sustaining habitats which require limited management. This concept was originally developed for use in mangrove ecosystems, and is understandably controversial, as it markedly diverges from other protection approaches that assign human use a minor priority or exclude it. However, the advantage of ED lies within the considered implementation of these designed ecosystems, thus preserving human benefits from potential later disturbances. Here, we outline the concept of ED in tropical carbonate depositional systems and discuss potential applications to aid ecosystem services such as beach nourishment and protection of coastlines and reef islands at risk from environmental and climate change, CO_2_ sequestration, food production, and tourism. Biological carbonate sediment production is a crucial source of stability of reef islands and reef-rimmed coastlines. Careful implementation of designed carbonate depositional ecosystems could help counterbalance sea-level rise and manage documented erosion effects of coastal constructions. Importantly, adhering to the core ethos of ED, careful dynamic assessments which provide a balanced approach to maximizing ecosystem services (*e.g.,* carbonate production), should identify and avoid any potential damages to existing functioning ecosystems.

## Introduction

Humans have altered or ‘designed’ ecosystems for millennia to benefit from their services (Vitousek et al., 1997; Zimmer, 2018). Such interventions required forethought and planning (*e.g.*, seasonal planting and harvesting), and usually ongoing management (*e.g.*, removal of weeds and pests, use of fertilizers *etc.*) to yield the desired services. Ecosystem design (ED) is generally defined as (1) the implementation of novel ecosystems in degraded areas (Zimmer, 2018), or (2) the steering of existing ecosystems and their use, in order to sustain or improve ecosystem service-provisioning and ensure their sustainable use (Rode et al., 2016). In all cases, ED aims to intentionally create healthy and resilient ecosystems that provide ecosystem services as required by societies and local communities. Ideally, all efforts toward ED would incorporate sustainable practices, namely understanding and integrating economic development, characterizing the changing human needs and aspirations, and documenting the capacity of the environment to absorb the consequences of human activities (Hay & Mimura, 2006). Importantly, ED differs from restoration and conservation efforts in that it is intentionally centered around the needs of society, *i.e.,* on the services provided by ecosystems.

This is in sharp contrast to—and potentially conflicting with—conservation aims to protect ecosystems in their natural and unaltered states as a first priority, which in most cases means excluding human influence (Hockins et al., 2006). Restoration-based approaches to management seek to re-establish a prior, potentially pristine, historical state predating human degradation (Higgs et al., 2014). Recent coral reef restoration efforts have focused on mass-culturing of specific reef-building coral species to repopulate degraded reefs, allowing the recovery of framework complexity (Rinkevich, 2021). However, there are several rarely considered issues, which may influence perceptions of success surrounding outcomes of restoration approaches. First, one problem is the difficulty in defining the baseline in highly dynamic conditions (*e.g.*, recent transition from the Little Ice Age, which ended in the late 1800s, to modern climate; Perring, Standish & Hobbs, 2013; Abelson et al., 2016). Tropical oceans over the millennia have experienced pronounced climate change, and thus a decision needs to be taken whether to restore to an arbitrarily defined past (“original”) baseline, or to prepare the system to be resilient to future predictions. This phenomenon of shifting baselines, however, is not only caused by climate change but also by anthropogenic disturbances such as fisheries that for decades or even centuries systematically have removed large predators or large herbivores, regionally leading to functional extinction (Jackson et al., 2001). A better understanding of the dynamics of carbonate ecosystems, including their ecological trajectories and climate legacies, could help to better predict their resilience, adaptation potential and limits. Second, coral reef restoration has largely centered on corals only, neglecting many species which contribute to the biodiversity and functioning of reef systems (Rinkevich, 2021). Third, a large range of methodologies defining success hinders its evaluation (Boström-Einarsson et al., 2020; Hein et al., 2020). Lastly, restoration efforts often focus on the ecosystem itself rather than on the services provided by this ecosystems as for example local fisheries pose a challenge in densely populated regions with high dependence on these services (White et al., 2014).

The benefit of viewing human intervention efforts in marine systems from the ED perspective is that it offers an alternative framework centering around considerations to (re)create ecosystems that provide services used by humans and are robust towards the use of these services under predicted environmental conditions (targeted resilience). Of particular importance to the ED framework are explicit criteria for defining success early on in the approach, as well as continuing reassessment of milestones. In contrast, current conservation efforts have focused on a primary goal of preserving the intrinsic value of biodiversity (Kareiva & Marvier, 2012). However, Ross et al. (2015) describe a recent general shift in restoration efforts, away from attempts to recreate historic ecosystems toward the restoration of quantifiable ecosystem services (*e.g.*, carbon sequestration, nitrogen retention, number of visitors) and even including the enhancement of defined and desired services. Nevertheless, the concept of ED is often met with resistance by conservation and restoration practitioners and decision makers (Murcia et al., 2014), due to confounding historical, social, cultural, political, aesthetical, economic, and ethical aspects. We recognize that any modern approach of ecosystem intervention will have to be negotiated among governments, local users and stakeholders, opposing and competing interest groups, *etc.*, in the light of the ecosystem services required locally, regionally and globally.

## Rationale

In this conceptual perspective, we outline the possibilities of ED within tropical carbonate depositional systems. We propose that the approach of ED can form a valuable part of the sustainable development of tropical coastlines. However, we accept that it will not be appropriate in every situation. Thus, this perspective paper is aimed at scientists, conservationists, restoration managers, and developers who are open to unconventional approaches.

Tropical coral reefs and related habitats provide valuable ecosystem services to millions globally, through the construction of calcium carbonate habitats by ecosystem engineers including scleractinian corals (Idjadi & Edmunds, 2006), calcifying green algae (McNeil et al., 2016), and large benthic foraminifera (LBF; single celled photo-symbiotic protists, see references in Narayan et al., 2021). With the geological view on time-dependent dynamics, we draw from our understanding of past sea-level changes in carbonate deposition to understand how future ED efforts may aid in preservation of carbonate depositional systems in future climate change scenarios. We discuss ecosystem service provision by carbonate depositional systems focusing on shoreline protection and maintenance, food resources, tourism, and climate mitigation. Subsequently, we highlight how these ecosystem services may be combined with ED techniques to better—and sustainably—use the services provided by these systems. This is especially relevant in light of rapid climate change degrading modern carbonate depositional systems, where, *e.g.*, increased sea surface temperatures may lead to bleaching and mortality (Schmidt et al., 2011; Stuhr et al., 2017; Sully et al., 2019). Lastly, we highlight the current gaps in knowledge that need to be targeted for a successful implementation of ED in carbonate producing habitats, as well as practical approaches and considerations prior to embarking on this endeavor.

## Survey Methodology

This conceptual perspective paper is based on published literature. As ED is a relatively new approach, conceptualized by Zimmer (2018), and never before used for carbonate depositional systems and reefs, we base our perspective paper on a synthesis of literature on ED in mangroves, restoration and conservation literature for modern carbonate depositional systems and in particular coral reefs, their reaction to sea-level fluctuations, and on the geological record of such carbonate depositional systems. No previous review of the topic exists as this is a novel field.

To assure a comprehensive and unbiased coverage of literature, we searched the following literature databases: Research Gate, ISI Web of Science, and Google Scholar. The search terms used included: restoration, conservation, reconstruction, ecosystem design, ecosystem loss, coral, *Halimeda*, red alga, foraminifer, sea-level, drowning, small islands, reef islands, reef health, growth rate, sedimentation rate.

Articles were included when they provided information not covered by previous literature (priority principle) or included in comprehensive review papers (principle to keep reference list as concise as possible).

### Reef islands and carbonate depositional systems

From a geological perspective, carbonate depositional systems such as tropical reefs are exposed to constant change through time, while shorter-term ecological perspectives provide a different perception of change. This difference in perspectives of time can influence our grasp of the functioning of ecosystems and how they may react to human induced disturbance. Numerous studies have documented the world-wide decline of tropical coastal ecosystems (*e.g.*, coral reefs, seagrass meadows, mangroves) and a loss of biodiversity (Hughes et al., 2018). A common theme which emerges is that reducing emissions is the only viable solution to mitigate the impacts of climate change (Bruno, Côté & Toth, 2019). However, this fails to recognize alternative and supplementary ways to safeguard such ecosystems (Abelson, 2019), and that modern reefs, having established after the last Ice Age, persisted during fluctuating climate in the roughly 10k yrs of the Holocene (Camoin & Webster, 2015).

The challenges associated with using a relatively arbitrary but recent baseline as the aim of a restoration project are well illustrated by data from the Caribbean, where coral reef communities have changed enormously since the 1960s/70s (Gardner et al., 2003). Here, populations of *Acropora* and *Orbicella* species, which are responsible for the majority of Holocene reef growth, have declined due to bleaching and disease (Aronson & Precht, 2001; Baker, Glynn & Riegl, 2008; Eakin et al., 2010), and this has led to the development of novel coral assemblages (Green, Edmunds & Carpenter, 2008; Toth et al., 2019), potentially altering reef function (Alvarez-Filip et al., 2013; Perry et al., 2015a; González-Barrios, Cabral-Tena & Alvarez-Filip, 2021). Caribbean coral reefs have not recovered from the many and widespread disturbances catalogued since the 1980s and therefore it is possible that they have stabilized in an alternate state (Knowlton, 1992), which would oppose attempts to restore a previous state. Equally, in other regions, research has shown that disturbance events can lead to changed coral community dominance after coral cover has recovered to pre-decline levels (Pacific: Adjeroud et al., 2009; Indian Ocean: McClanahan, 2014). These novel benthic communities may provide different ecosystem services or alter the regime providing existing ones, hence management of marine systems would benefit from considering the potential benefits accrued from different communities (Kuffner & Toth, 2016).

Recently, the idea of drowning reefs and sinking islands and atolls due to global sea level rise (SLR) has received a lot of public attention, leading to predictions that low-lying reef islands will become physically unstable and unsuitable for human populations (Kelman & West, 2009; Storlazzi et al., 2018). However, this is in strong contrast to the geological record, which shows that reef-rimmed carbonate depositional systems increase calcium carbonate production in response to SLR, given there is lateral space available for a lagoon (Pomar & Ward, 1995; Pomar, Ward & Green, 1996). High rates of tropical carbonate production are restricted to shallow water (<30 m) because light penetration decreases exponentially with water depth and many carbonate producers are photosynthetic (*e.g.*, algae) or photosymbiotic (*e.g.*, corals). Platform drowning occurs when the region of maximum carbonate productivity reaches water depths below the photic zone as a result of subsidence or sea-level rise (Schlager, 2005). Drowning has been documented from times when sea-level rose at rates an order of magnitude higher than those observed today (*e.g.*, 40–50 mm yr^−1^) during a late-deglacial melt-water pulse (Sanborn et al., 2017).

Small island states in particular in the Pacific have to cope with sea-level rise, and this awareness has led to a wide range of international programs to help adapt to the ongoing and expected changes (Freestone & Cicek, 2021). The common and persistent narrative of island loss and necessary exodus, however, has undermined adaptive planning in island nations (Barnett, 2017). Instead, local defending strategies have been implemented that might not be sustainable, but maladaptive, such as sea-walls, in particular when the building material is taken from the adjacent fringing reef (Hay & Mimura, 2006; Kench, 2012; Duvat, 2013). The assumption that reef islands are static landforms that drown as sea-level rises, is flawed, while in fact they are dynamic but persistent features that adjust to sea-level change (Webb & Kench, 2010). Remotely sensed data of the last four decades of the island nation of Tuvalu that is subject to twice as high sea-level rise as the global average (∼3.90 ± 0.4 mm yr^−1^) shows an increase of land area by 2.9% (the larger islands having grown, the smaller ones showing both, growth and erosion; (Kench, Ford & Owen, 2018). The physical stability of carbonate islands is dependent on the existence of carbonate producing ecosystems that build the foundation for continuous sediment delivery (Stoddart & Steers, 1977; Perry et al., 2011) and to grow with rising sea-level (Beetham & Kench, 2018; Masselink, Beetham & Kench, 2020). Island growth is an expression of increased sediment production during sea-level rise, an observation critical for the resilience of such islands, depending crucially on reef health.

The potential accumulation rate of many modern coral reefs and carbonate platforms (>8 mm yr^−1^ in branched coral facies, to <5 mm yr^−1^ in head coral facies on the GBR; Hopley, Smithers & Parnell, 2007), for detailed rates for specific settings and regions see (Montaggioni, 2005) is higher than recent (1.7 mm yr^−1^; 1901–2010) and current (3.2 mm yr^−1^; 1993–2012) rates of global relative SLR (Church et al., 2013). However, it should be noted that estimates of reef accretion potential in the western Atlantic and Indian Ocean suggest that reef degradation will mean that few coral reefs can keep pace with projected rates of SLR during this century (Perry et al., 2018). The upper limit of marine carbonate accumulation is determined by physical constraints such as the base of storm waves, limiting upward growth of non-framework carbonate habitats such as *Halimeda* bioherms or foraminiferal carbonate ramps that are only weakly stabilized, while framework building carbonates formed by corals and crustose coralline red algae (CCA) can grow to the sea-surface also in high-energy regimes (Eberli & Grammer, 1999). Accumulation above sea-level, *i.e.,* build-up of islands, mainly depends on the energy of storm waves and wind carrying marine carbonate sediment to beaches and further inland (wash-over deposits, storm deposits, dunes; (Kench & Mann, 2017).

Modern reef island sediment is typically composed of corals, calcareous green algae (*e.g.*, *Halimeda)*, CCA, foraminifera, molluscs, echinoids, and other carbonate grains (Stoddart & Steers, 1977; Perry et al., 2011; Janßen et al., 2017; Woodroffe et al., 2017). *Halimeda* species and LBF are among the most important carbonate sediment producers globally (Fujita et al., 2009; Fujita et al., 2016; Garnier, 2012; Doo et al., 2017; Narayan et al., 2021). In the modern GBR, *Halimeda* bioherms significantly exceed adjacent coral reefs in area and production (McNeil et al., 2016). *Halimeda* produce carbonate skeletons of primarily sand-grade size (0.06–2.00 mm) and thus are directly suitable for island build-up and beach nourishment, while larger skeletons and frameworks are converted to sand-sized grains by degrading processes such as bioerosion and wave action. In the Maldives, the majority of island building sediment is produced from the excretory byproduct of parrotfish feeding activity (Perry et al., 2015b). In densely populated regions of the tropics overfishing can result in a decrease of this form of sediment generation, and can also lead to increased competition for space as fleshy and turf algae benefit from the removal of their predators, affecting coral recruitment (Steneck, Arnold & Mumby, 2014). Accordingly, one direct avenue for a targeted increase in reef sediment production and subsequent stabilization of shorelines could focus on triggering the proliferation of reef organisms that produce primarily sand- to granule sized sediment particles. However, increasing sediment production from bioeroders may reduce the three-dimensional framework complexity of reef habitats and have knock-on effects on reef function as shown from east African reefs where overfishing has led to increased urchin populations, bioerosion and reduced complexity (McClanahan & Muthiga, 1988; Humphries, McClanahan & McQuaid, 2020).

### Ecosystem service provision by carbonate depositional systems

**Figure 1:**
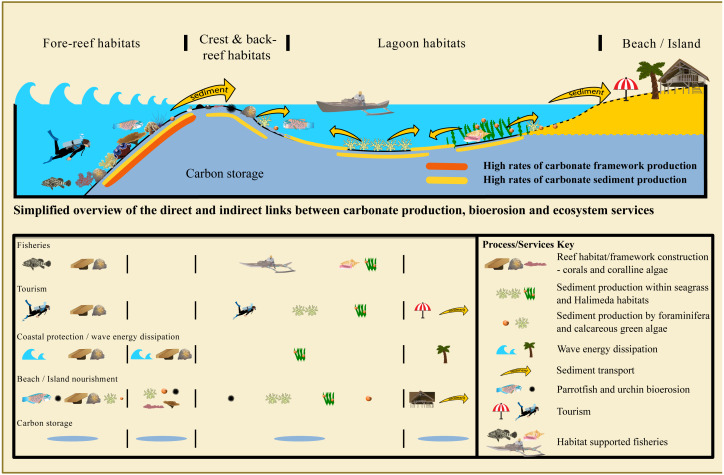
Theoretical composite distribution of carbonate framework and sediment producers, transport and depositional processes common to tropical coral reef ecosystems and atolls and their associated ecosystem services. Fore-reef habitats are characterized by high abundance and diversity of reef-building corals which provide structural complex habitats for fish, attracts dive tourism and generates carbonate sediment through bioerosion, *e.g.*, by parrotfish or sea urchins. Along with the reef crest, where the carbonate framework is supported by crustose coralline algae and other calcareous encrusters, it dissipates wave energy and reduces coastal erosion. Back-reef and lagoonal habitats are characterized by the dominance of carbonate sediment producers such as calcifying green algae (*e.g.*, *Halimeda*) and benthic foraminifera and epilithic calcareous encrusters, often associated with seagrass habitats. These contribute to beach or island nourishment, and play an important role in the development/sustainability of habitats whichprovide important resources for fisheries and tourism. The produced carbonate sediment is accumulated in low-energy areas or transported to the shore where it creates islands and beaches, which are crucial for coastal/island communities and tourism. The consolidation and burial of carbonates contributes to long-term carbon sinks.

Carbonate depositional systems provide a range of ecosystem services that could potentially be enhanced by ED. This includes direct services from habitats (*e.g.*, food resources and dive tourism) as well as indirect, regulatory benefits such as a reef’s breakwater function, reducing wave energy and protecting coastlines. Additionally, the deposition of carbonate sediment stabilizes shorelines and allows the development of natural beach/island nourishment regimes. Hence, there is a physical connectivity driven by the production of calcium carbonate, that could be called carbonate connectivity, and which underpins the development of the spatial extent of different marine habitats and landscapes like beaches and reef islands (Fig. 1).

**Figure 2:**
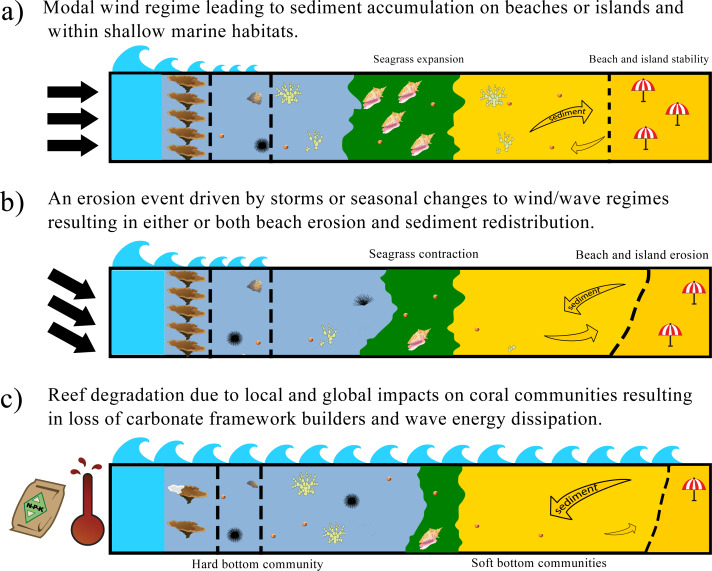
Carbonate framework and sediment production, transport and accumulation altered by variability in the physical system. (A) Fairweather conditions with high coral cover and framework complexity on fore-reef habitats and reef crest dissipate wave energy allowing the development and expansion of lagoonal habitats (*e.g.*, seagrass). These conditions provide a supply of carbonate sediment for beach and island nourishment; (B) episodic or seasonal natural disturbance events may contract lagoonal habitats potentially reducing carbonate sediment production, and redistribute sediment or increase erosion along sedimentary landforms; (C) anthropogenic disturbances due to local (*e.g.*, increased nutrient input) or global (*e.g.*, bleaching) impacts lead to degradation of various reef habitats, especially the reduction of fore-reef and reef crest carbonate framework, which decreases wave energy dissipation, potentially altering sediment production regimes in lagoons and increasing shoreline erosion.

### Shoreline protection and nourishment

Shoreline protection and the nourishment of beaches and islands with sediment are regulatory ecosystem services (Woodhead et al., 2019) that along tropical coastlines are dependent on biological carbonate production, deposition and accumulation (Perry et al., 2011), except where terrestrial sediment input *via* rivers or dust is dominant. Reef frameworks that act as breakwaters also play an important role in shoreline protection. To fully appreciate how carbonate production in its different forms (sediment and framework) combines to provide these regulatory ecosystem services, it is important to consider seascape development during the Holocene. Most modern coral reefs have grown to the sea-surface during the Holocene, and in doing so created sedimentary environments behind the reef crest that are protected from wave energy (Figs. 1 and 2). This protection comes from both the depth of the reef and its structural complexity, with more complex reefs dissipating more wave energy (Graham & Nash, 2013; Monismith et al., 2015; Harris et al., 2018). Over time, reef associated habitats (*e.g.*, seagrass beds) initiated or expanded in association with the development of these sedimentary environments and as a result of carbonate sediment produced in the reef and associated habitats (Fig. 2A). Hence biogenic carbonate production as framework (corals, CCA) or sediment (*Halimeda*, *Penicillus*, LBF), including physical and biological erosion, leads to the initiation, development and maintenance of sedimentary habitats and also sedimentary landforms (Kench, McLean & Nichol, 2005; Ford & Kench, 2012; Gischler et al., 2013; Morgan & Kench, 2016). Sediment produced on the reef is transported to other areas *via* wave energy dynamics and currents (Fig. 1), which are both influenced by the presence of a reef and its benthic community. Many habitats and landforms (including islands) in the lee of coral reefs could not establish without the presence of the reef, and their sustainability is dependent on either or both, wave energy dissipation and sediment supply (Fig. 2). Hence, their ecosystem services are also causally dependent on and underpinned by carbonate production.

Carbonate beaches, dunes and islands show a dynamic balance between accreting and eroding forces where composed of unconsolidated and mobile sediment (Figs. 2A, 2B). Periods of accretion and erosion can be cyclical, often occurring seasonally (Kench & Brander, 2006) and potentially redistributing sediment in response to wind-driven currents (Fig. 2B) or in response to episodic disturbance events such as hurricanes and tsunamis (Kench et al., 2008; Mann & Westphal, 2016). However, ultimately all systems require a source of new sediment such as *Halimeda* bioherms, sea-grass meadows, and coral reefs. Changes to habitat extent or health will alter the populations of sediment producers and therefore the quantities and the type of sediment available to nourish beaches and islands (Li, Jones & Blanchon, 1997). However, bleaching, ocean acidification, and eutrophication of coastal waters are affecting populations of carbonate sediment producers (Fig. 2C) and thereby sediment nourishment regimes (Cockey, Hallock & Lidz, 1996; Hallock, 2000; Castro-Sanguino, Bozec & Mumby, 2020).

Recent research into beach and island nourishment regimes in carbonate depositional systems has highlighted the importance of different species, *e.g.*, parrotfish in the Maldives (Morgan & Kench, 2016; Perry et al., 2020), foraminifera on the GBR (Doo, Hamylton & Byrne, 2012; Doo et al., 2017), and molluscs in the Gulf of Mexico (Kasper-Zubillaga et al., 2017). Hence, it is likely that sediment nourishment regimes are unique to individual beaches and islands with a combination of local production and physical transport mechanisms (waves energy, currents, tides) shaping these dynamic systems. Few studies have explored the connectivity between ecological communities and carbonate sediment provision, however, this is essential for understanding how to design carbonate depositional systems which provide the intended ecosystem services. Attempts to model this complexity are at an early stage (East et al., 2020; Tuck et al., 2021) but as our understanding improves particularly at a local level, societies can begin to accomodate future changes in sediment regimes by incorporating biogenic nourishment in their management plans.

### Food resources and tourism

Fisheries and tourism are examples of provisioning and cultural ecosystem services gained from tropical marine ecosystems (Moberg & Rönnbäck, 2003; Woodhead et al., 2019). For instance, coral reefs are among the most biodiverse ecosystems globally and therefore an attraction for tourists and the target of fisheries. Coastal societies are reliant on ecosystem services provided by these ecosystems, whether they are deemed healthy or degraded. In many regions, fisheries act as the main food resource to coastal communities and along with tourism can provide employment underpinning local economies. The direct exploitation of both, fisheries resources and tourism, are complexly linked to the deposition of calcium carbonate. Co-evolution of neighboring habitats (*e.g.*, coral reefs, seagrass and mangroves) has allowed the development of connectivity between them leading to mutual benefits and improved ecosystem service provision (Mumby et al., 2004; Guannel et al., 2016; Olds et al., 2016).

On coral reefs, carbonate deposition builds the complex three-dimensional structure that supports both, the biodiversity and biomass of these ecosystems; more complex reefs support larger biomass and greater species diversity (Gratwicke & Speight, 2005; Newman et al., 2015). Additionally, biodiversity is widely considered to be important in the functioning of these ecosystems (Alvarez-Filip et al., 2013) with changes in species assemblages leading to altered function (Perry et al., 2015a; Perry et al., 2020). By growing to the surface, coral reefs create depositional environments where seagrass and other habitats can develop or expand biodiversity and biomass, which may be exploited as a fishery resource. For example, the queen conch (*Strombus gigas*) that spends much of its complex life cycle in lagoonal habitats (Stoner, 2003), is a common fishery throughout the Caribbean. Likewise, many reef fish species use nursery habitats behind the reef and undergo ontogenetic migrations as they mature (Nagelkerken et al., 2000; Mumby et al., 2004).

Natural assets like coral reefs and carbonate beaches may attract millions of visitors annually, and the economic benefits associated are dependent on the deposition of calcium carbonate (Fig. 1). As with fisheries, structurally complex habitats are important to the biodiversity and abundance of the marine organisms that attract snorkelers and scuba divers. Hence, the management of coral reefs to maintain structurally complex habitats and ultimately biodiversity and biomass must consider carbonate production and bioerosion (Perry, Spencer & Kench, 2008; Brandl et al., 2019; González-Barrios, Cabral-Tena & Alvarez-Filip, 2021).

### Carbon sequestration

Climate change mitigation strategies have considered the potential for certain ecosystems to be carbon sinks, sequestering carbon from the atmosphere. While most studies have focused on temperate marine regions, and in particular primary producers such as kelp forests or seagrass beds (Ricart et al., 2020), the efficacy of these carbon sinks for longer-term (greater than decadal time frame) sequestration is unclear. When considering carbon sequestration processes in tropical marine ecosystems, organic sequestration is primarily linked to biomass accumulation in primary producers such as mangroves (Alongi, 2014) and seagrasses which store organic carbon in leaves and rhizomes (Koch et al., 2013). While these processes remain relatively well quantified, recent studies have shown the potential for rapid declines in stored carbon due to climate-induced marine heat waves (Arias-Ortiz et al., 2018). In carbonate depositional systems such as tropical reefs, large quantities of carbon are stored as inorganic CaCO_3_ and potentially removed from the carbon pool for up to many millions of years. The exact time-span is dependent on a variety of physical, chemical and biological processes which require investigation for different habitats. However, a significant debate remains as to whether calcification mechanisms are actually net sources or sinks of carbon, as this depends on the process in which calcification occurs (McConnaughey & Whelan, 1997). While the process of calcification generally is accepted to emit CO_2_, it is unknown how the organismal processes of individuals scale to complex ecosystem processes that include primary producers. For example, there is uncertainty regarding whether seagrass meadows (including the associated calcifying flora and fauna such as red algae and foraminifera) have the potential to be CO_2_ sequesters (*e.g.*, *Posidonia* meadows; (Serrano et al., 2012). In these interactions between primary producers and calcifiers (*e.g.*, epiphytes on seagrasses), intricate feedbacks between photosynthesis and calcification processes take place (Doo et al., 2020; Brandano et al., 2016) that are worth further investigation, and in particular the mechanisms in which ecologically relevant scenarios of species interactions could be harnessed to promote carbon sequestration in tropical marine ecosystems.

### Future directions and considerations of ED in carbonate depositional systems

ED offers significant potential for achieving current conservation goals in addition to improving or stabilizing ecosystem services crucial to humans. However, the challenges associated with understanding the natural functioning of complex ecosystems, their connectivity and feedback from resource use in these coupled human-natural systems are not trivial. Practical approaches to harmonizing ecosystem service provision and sustainability are rare. The term ecomimicry has been used to describe a strategy for developing and managing cultural landscapes based on ecological knowledge and with the goal of sustaining key ecosystem services; an approach that has been traced back to Hawai’ian indigenous practices for balancing various services (Winter et al., 2020).

On a large scale, the Red Sea Project, situated on the Al Wajh carbonate platform (Bruckner et al., 2012; Petrovic et al., 2021), attempts to develop an environmentally friendly tourist destination while maintaining biologically diverse habitats such as seagrass meadows, mangroves, and coral reefs. By embracing the conservation of the region as a primary goal for all stakeholders and using iterative rounds of marine spatial planning and workshops, the project aims to achieve positive conservation outcomes with clearly defined conservation (Chalastani et al., 2020). Additionally, the project includes elements of future-proofing the coral reefs against increasing occurrences of marine heat waves (Osman et al., 2018) by planning for multiple coral nurseries with the potential to interbreed coral species to develop thermal resilience (Chalastani et al., 2020). While the approach of selective breeding and outplanting to improve thermal tolerance and long-term resilience is still in its infancy and requires substantial ecological risk–benefit assessments (Voolstra et al., 2021; Kleypas et al., 2021) the Red Sea project offers the chance to acquire data on the success of such approaches.

Creating novel or adapting existing ecosystems to provide targeted services requires the definition of the targets and a sound knowledge of ecosystem functions and processes to avoid unintended consequences. Vague aims like increasing biodiversity are insufficient as a defining goal, unless this is coupled with a measurable enhancement of an ecosystem service *e.g.*, increasing fish diversity for diving tourism. Some highly productive, eutrophic, carbonate depositional ecosystems are naturally characterized by low biodiversity (Westphal, Halfar & Freiwald, 2010), and increasing biodiversity would be an intervention that requires a specific reason. Zimmer (2018) pointed out that the natural sciences’ role in ED includes the provision of information in ecosystems and their functioning, ideally feeding into a tool kit for decision makers. The idea of a tool kit has also been explored by Frias-Torres, Montoya-Maya & Shah (2018) for reef restoration as a learning system that will improve with experience. ED projects will need to (i) monitor the development of ecosystems, the services provided and how these meet project goals (ii) allow for continued intervention (to be kept minimal) (iii) adapt project goals as societal requirements change or if the ecosystem does not stabilize sufficiently in the targeted direction.

A range of potential approaches to ED within carbonate depositional systems are described in Table 1. In the following we elaborate on the interventions, potential benefits, and knowledge gaps.

**Table 1:** Potential approaches for designing shallow marine carbonate systems to target specific ecosystem services and the knowledge gaps associated with their implementation.

Desired Ecosystem Services	ED interventions	Knowledge gaps
**Food Resources and Tourism**		
Sustainable reef-related fisheries (including economically important organisms such as molluscs, holothurians, *etc.*)	Lagoon habitat expansion for targeted fisheries Targeted use of artificial frameworks, coral outplanting to improve reef structural complexity in local habitats Mariculture of seagrass or corals to provide suitable habitat for target species (*e.g.,* conchs, juvenile fish)	Associated foundation species required to initiate sustainable ecosystem for fisheries Limited understanding of the habitat and organismal connectivity; ecology, biogeochemical cycling, carbonate connectivity *etc.* Species-interactions (competition *vs* mutualism) and optimum conditions for mariculture of designed habitats
High biodiversity and seascape morphology for recreational underwater tourism	Construction of small-scale reef structures or artificial reefs that support increasing reef biodiversity and biomass Increasing resilience to environmental stress through molecular interventions (introduced symbiont shuffling, probiotics, assisted evolution)	Potential physicochemical impacts (hydrodynamics, structural complexity) on existing communities from implementing artificial reefs Potential side effects of interventions from introducing non-native species or genes. Understanding trade-offs with other biological functions of these organisms is crucial
Attractive vacation beaches	Mass production of sediments for beaches through mariculture of carbonate sediment producers (*e.g.,* “living sand” foraminifera) Management of bioeroder populations for optimal sediment production	Limited understanding of species-specific carbonate sediment production rates, sediment transport dynamics, and the influence of habitat structural complexity Considered management that weighs the risk of damaging existing carbonate framework with benefits of generating additional sediments for beaches
**Shoreline Protection and Nourishment**		
Shoreline protection through wave energy dissipation	Artificial frameworks which promote coral or calcareous encruster settlement; coral outplanting Electrolysis-mediated carbonate precipitation on reefs to augment loss of wave energy dissipation from reef degradation	Potential for promoting reef framework development through culturing encrusting organisms such as coralline red algae, foraminifera Feasibility of scaling
Reef island nourishment	Mass production of sediments for beaches through mariculture of carbonate sediment producers (*e.g.,* “living sand” foraminifera) Management of bioeroder populations for optimal sediment production	Limited understanding of species-specific carbonate sediment production rates, sediment transport dynamics, and the influence of habitat structural complexity. Considered management that weighs the risk of damaging existing carbonate framework with benefits of generating additional sediments for beaches
Management of sediment transport pathways	Manipulating current channels from carbonate production (sources) to intended deposition (sink)	Quantifying hydrodynamic effects, side effects of reducing sediment export to deeper water (slope stability)
**Carbon Sequestration**		
Medium- to long-term storage of carbon	Increasing carbon sequestration through exploiting organic-inorganic feedbacks (photosynthesis-calcification)	Limited understanding of the temporal scaling in carbon sequestration, as well as optimal co-culturing pathways and source-to-sink dynamics for longer-term carbon burial.

### Interventions supporting shoreline protection and nourishment

As described above, shoreline protection and nourishment ecosystem services in tropical carbonate depositional systems are dependent on carbonate framework and sediment production. Hence, ED interventions should target structural complexity of reefs and growth or sediment production regimes. Transplanting corals from productive systems or nurseries should be considered toward the goal of maintaining reef structural complexity. Target species would be robust branching corals which do well in shallow high energy environments. The maintenance of structural complexity using artificial structures should also be considered where these promote the settlement of CCA or corals and act as a foundation for the development of a functioning ecosystem. CCA play a critical role in reef growth as they provide substrate for coral settlement, stabilize unconsolidated coral rubble and strengthen dead coral framework. Their increased diversity is concurrent with a transition in reef morphology during the Late Miocene, when coral reefs, encrusted by CCA, climbed up to the sea surface to build a rigid, wave-resistant framework (Pomar et al., 2017). CCA potentially provide opportunities for maintaining reef function in the wake of mass coral mortality events. Electrodeposition (Hilbertz, 1979), although energy intensive, could provide opportunities to maintain reef growth or boost coral growth in certain situations. The technique encourages the deposition of calcium carbonate, and experiments which investigate the use of this technology to deliver specific ecosystem services should be encouraged.

The mariculture of non-coral carbonate producers with the purpose of producing or stabilizing carbonate sediment is as yet underexplored. Hosono et al. (2014) examined the feasibility of mass-culturing the LBF *Baculogypsina sphaerulata* with a long-term view of developing resilience to SLR for low lying islands dependent on this species as a sediment source. Evidence from the geological record shows that during the middle to late Holocene, increased rates of foraminiferal sediment production triggered the development and extension of some of the largest coral islands in the Indian and Pacific oceans (Kench, Owen & Ford, 2014; Dawson, Smithers & Hua, 2014; Yasukochi et al., 2014; Kane & Fletcher, 2020; Kench et al., 2020). Rates of carbonate sediment production by some modern benthic foraminifera have been reported to be high (0.15–2.8 kg CaCO_3_ m^−2^ yr^−1^; Hallock, 1981; Langer, Silk & Lipps, 1997; Langer, 2008; Narayan et al., 2021). Hence, mass-culturing LBF within appropriate locations may mitigate negative impacts of future SLR on shoreline stability (Hosono et al., 2014).

Calcareous green algae are among the highest contributors to sediment production in lagoon and back reef environments with some of the most productive species belonging to the *Halimeda* and *Penicillus* genera (Neumann & Land, 1975; Wefer, 1980). Hence the mass-culture of such species has high potential for ecosystem design projects requiring large quantities of sediment. However, many species produce very fine mud-grade material which may not provide the ideal sediment type to nourish beaches or islands. Sediment size is important because of differential transport and preservation pathways (Folk & Robles, 1964; Ford & Kench, 2012). *Halimeda* species produce more sand-grade material and calcify rapidly, adding new segments every 1–2 days, and production estimates for natural populations can be very high (*e.g.*, 1.0–2.5 kg CaCO_3_ m^−2^ yr^−1^; (Castro-Sanguino, Bozec & Mumby, 2020). *Halimeda* species may also reproduce asexually by fragmentation, potentially providing a relatively straightforward mechanism to scale up production in mariculture settings. Additionally, they may be more resilient to global warming than other organisms (*e.g.*, LBF bleach and stop calcifying at temperatures above their local adaptation thresholds; Schmidt et al., 2011; Stuhr et al., 2017), while increased temperatures do not appear to inhibit *Halimeda* growth and may mitigate the effects of ocean acidification (Campbell et al., 2016). However, the overall percentage of intact *Halimeda* segments within the highly energetic swash zone along many shorelines is below 10% of the total sediment composition (Perry, 2000; Janßen et al., 2017; Perry et al., 2020), likely resulting from the low durability of segments during sediment transport and reworking.

Production rates of carbonate secreting organisms are still poorly constrained in different habitats and under different environmental conditions. Any ED project seeking to boost sediment production would have to investigate local carbonate production rates and develop an understanding of connectivity and sediment transport dynamics (see Fig. 2).

Beyond enhancing carbonate productivity, the manipulation of sediment transport pathways to beaches and islands could be considered as part of ED projects. The alteration of transport pathways could potentially be achieved by incorporating information on directions of mean currents, waves or periodic storm events into spatial planning efforts. Planning hydrological interventions requires deep understanding of the current system and wave climate, supported by modelling approaches. For example, it has been shown that on fringing reefs lower frequency, infragravity wave energy is important for bedload transport of reef-derived sediment across the reef flat and lagoon to the shoreline (Rosenberger et al., 2020). Suspended sediment transport seems to be controlled by mean currents on the reef flat, although sediment suspension is triggered through swell- and infragravity waves (Pomeroy et al., 2021). Notably, such mechanisms that induce sediment transport from the reef to the shoreline are likely to alter with future sea-level rise and related changes in reef flat topography (Bramante et al., 2020; Pomeroy et al., 2021). Accordingly, divergent patterns of sediment redistribution might induce unintended shoreline erosion and deposition, as seen in development projects in the waters of Abu Dhabi (Van Lavieren et al. 2011). Additionally, when implementing larger structures for field-based mass-culturing approaches or hard bottom substrate provisioning, targeted manipulation of sediment transport pathways through morphological adaptations of such structures should be considered. Reduction of flow velocities and enhanced trapping facilitates the settlement of sediments, while narrow channels or turbulence increase transport distances.

The effect of bioerosion on sediment production needs to be included in considerations on ED of carbonate depositional systems. Different organism groups produce different quantities of erosional sediment, and a balance with production is crucial. A large portion of sediment is produced from coral reef framework by parrotfish and urchins (Perry et al., 2012) and somewhat less by bioeroding sponges such as *Cliona* spp. (Murphy et al., 2016; De Bakker et al., 2018). It is observed that damage of reefs from bleaching or bioerosion events (*e.g.*, crown of thorns) are accompanied or followed by pulses of high sediment supply to beaches and islands (Perry et al., 2020). This is usually at the expense of reef framework stability. With parrotfish and urchins, larger adults erode relatively more as they feed (Bruggemann et al., 1996; Perry et al., 2012), and therefore managing their populations may be critically important to influencing both, sediment generation and framework erosion (Fig. 1). Non-coral hardgrounds could be explored for increasing sediment production by farming urchins in pens on hardground habitats to generate sediment.

### Interventions supporting food resources and tourism

Generally, the requirements for the provision of food and tourism-related services are similar as for coastal protection in reefs and related ecosystems in that they rely on carbonate production to build structurally complex habitats. The ongoing loss of structural complexity on many reefs negatively influences biodiversity and biomass, thus reducing food provision and the reef’s attractiveness to divers and snorkelers. ED approaches may help to increase those ecosystem services by creating new structurally complex habitat and potentially a self-sustaining ecosystem with reduced ongoing maintenance requirements. ED employing carbonates could potentially be used to expand nursery habitats, or the area available for extensive sea-farming, *e.g.*, *Strombus gigas* or sea cucumber fisheries.

While overall costs of restoration efforts are a necessary consideration, approaches to designing coral reef ecosystems and increasing structural complexity to maintain or increase biodiversity are still very experimental and expensive, and often have no clear assessment of the consequences of action. Artificial reefs utilizing sunken ships or other structures create novel habitats which may provide a tourist attraction, but bear little resemblance to typical coral reef ecosystems (Walker & Schlacher, 2014). However, it may be possible to use artificial structures to improve or encourage coral settlement (Suzuki et al., 2011), thereby boosting coral populations and ultimately reef complexity, biodiversity and biomass. Traditional attempts at boosting coral cover have focused on directly out-planting corals, with limited success because of high mortality rates, and because of the effort and costs involved when applying to large areas. Hence, designing out-planting strategies which create ‘stepping-stone’ populations of adult broadcast spawning species may benefit the recovery of reefs regionally (Kuffner et al., 2020). Moreover, it should be considered that reef-associated habitats, like seagrass beds or nearby terrestrial ecosystems, also influence the productivity of coral reefs through ecological connectivity (Harborne et al., 2006; Graham et al., 2018). Active interventions in such connected ecosystems (*e.g.*, reforestation or rat eradications to restore seabird populations and thereby natural nutrient subsidies; (Benkwitt et al., 2021) may therefore offer further opportunities to boost reef productivity and thus related ecosystem services.

Ideas around strengthening coral resilience to bleaching through interventions using probiotic approaches (Rosado et al., 2019), by exchanging photosymbionts (Baker, 2003), or other approaches to assisted evolution (Van Oppen et al., 2017; Buerger et al., 2020) are being discussed, in many cases controversially, as they are interventions in the natural spectrum of species, and potential trade-offs remain unknown. However, these potential interventions could maintain healthy populations of corals as global warming continues and the research should be pursued (Kleypas et al., 2021). Attitudes of the general public, stakeholders, institutions and governments on whether this type of intervention is justifiable or not in the face of reef loss and the loss of ecosystem services needs to be assessed when practical solutions become available.

### Interventions supporting carbon sequestration

Carbonate depositional systems have traditionally not been thought of as areas that can provide medium- to long-term carbon storage potential. However, long-term carbon storage is seen in many rock formations (*e.g.*, the Triassic Dolomites in Italy), leading to the conclusion that this is possible. Recent work on tropical carbonate depositional systems on carbon storage potential has focused on shorter-term (seasonal, annual) temporal dynamics in which seagrass meadows are able to store carbon in complex root rhizomes and leaves. Calcareous epiphytes (foraminifera, red algae, bryozoans), which reside in seagrass ecosystems, have the potential to produce high carbonate loads that add to the reef-scale carbonate budget (Perry & Beavington-Penney, 2005). Coral reefs sequester carbon through carbonate precipitation by corals, CCA and other calcifiers. However, many aspects of systems that produce carbonate skeletons with higher preservation potential remain to be understood to further exploit their carbon sequestration potential. Specifically, understanding the feedback dynamics between organic and inorganic carbon sequestration has been a topic of intense interest (Brandano et al., 2016). However, most conceptual models of carbon sequestration of coral reefs simulate either only living corals (Cyronak et al., 2018), or only calcareous sediments (Eyre et al., 2018). The medium- and long-term budget of the atmospheric CO_2_ contribution related to calcium carbonate precipitation is still being debated (Macreadie et al., 2017). Engineering has the potential to aid in such processes such as electrolysis-stimulated precipitation of calcium carbonate that could support the strengthening of framework or cementing loose sediments (Rau, 2009). Understanding existing carbon flow (both inorganic and organic) through carbonate depositional systems is crucially needed to better characterize reefs. Furthermore, once these processes are determined, local site-based information of source-to-sink dynamics are needed for spatial planning and design of these areas to ensure produced carbonates are stored in intended locations (*e.g.*, renourish beaches and coastlines).

## Conclusions

Most of the approaches discussed above are not new and not inherently coupled to ED. Rather, we place them in the context of ED as a framework for prioritizing, planning and assessing interventions for altering or manipulating (usually degraded) ecosystems. At its core, ED is a knowledge-based approach to the management of socio-ecological systems, with a focus on maintaining ecosystem services without endangering the ecosystem’s natural functioning. Hence, an important consideration for any ED approach is what services may be reasonably extracted from an ecosystem, and whether current societal needs can be met while maintaining ecosystem functioning. Obviously, a high level of knowledge is required on the effects and side-effects of such interventions, coupled with ethical discussions on the acceptance of the introduction of foreign or manipulated species. This highly relevant ethical discussion is beyond the scope of this paper, but we emphasize this as a condition sine qua non for any such approach. We deem it crucial to avoid any implementation of plantation-type intervention that impoverishes ecosystems with respect to their biodiversity and heterogeneity. Informed and responsible ED should inherently exclude this, but continued consciousness of this risk is necessary. From our point of view, an open and informed discussion on trade-offs, sacrifices and ethics has to accompany the implementation of ED approaches.

Knowledge gaps (Table 1) need to be narrowed to allow for informed decisions. If well informed, ED approaches have the advantage over conservation and restoration to be based on clearly defined goals and baselines, and on societal prioritization, thus allowing for decisions to be taken based on scientific advice offered, but grounded in and accounted for by a societal or political procedure. The approach puts human needs in the center and aims at preparing an ecosystem for being used without damaging it, that is, it couples resilience with sustainability, ideally offsetting the intervention costs with economic benefits. Having pointed to the limitations given by existing knowledge, ethics, and decision procedures, carbonate depositional systems appear promising for exploring the possibilities of ED. While fully acknowledging the influence of global change, a local approach offers a leverage to address at least some of the negative influences on ecosystem service provision in a tailor-shaped way, and including longer-term global influences in the considerations for a prospective vision and approach.

## Additional Information and Declarations

### Data Availability

The following information was supplied regarding data availability: The paper is a perspective that is based on published literature as cited in the article.
